# Assessment of North American Clinical Research Site Performance During the Start-up of Large Cardiovascular Clinical Trials

**DOI:** 10.1001/jamanetworkopen.2021.17963

**Published:** 2021-07-23

**Authors:** Akash Goyal, Tony Schibler, Brooke Alhanti, Karen L. Hannan, Christopher B. Granger, Michael A. Blazing, Renato D. Lopes, John H. Alexander, Eric D. Peterson, Sunil V. Rao, Jennifer B. Green, Matthew T. Roe, Tyrus Rorick, Lisa G. Berdan, Craig Reist, Kenneth W. Mahaffey, Robert A. Harrington, Robert M. Califf, Manesh R. Patel, Adrian F. Hernandez, W. Schuyler Jones

**Affiliations:** 1 The Ohio State University Wexner Medical Center, Division of Cardiovascular Medicine, Columbus; 2 Duke Clinical Research Institute, Duke University School of Medicine, Durham, North Carolina; 3 Verana Health, San Francisco, California; 4 2M Clinical, Arlington, Texas; 5 Stanford Center for Clinical Research, Department of Medicine, Stanford University, Stanford, California; 6 Verily Life Sciences and Google Health, San Francisco, California

## Abstract

**Importance:**

Randomized clinical trials (RCTs) are critical in advancing patient care, yet conducting such large-scale trials requires tremendous resources and coordination. Clinical site start-up performance metrics can provide insight into opportunities for improved trial efficiency but have not been well described.

**Objective:**

To measure the start-up time needed to reach prespecified milestones across sites in large cardiovascular RCTs in North America and to evaluate how these metrics vary by time and type of regulatory review process.

**Design, Setting, and Participants:**

This cohort study evaluated cardiovascular RCTs conducted from July 13, 2004, to February 1, 2017. The RCTs were coordinated by a single academic research organization, the Duke Clinical Research Institute. Nine consecutive trials with completed enrollment and publication of results in their target journal were studied. Data were analyzed from December 4, 2019, to January 11, 2021.

**Exposures:**

Year of trial enrollment initiation (2004-2007 vs 2008-2012) and use of a central vs local institutional review board (IRB).

**Main Outcomes and Measures:**

The primary outcome was the median start-up time (from study protocol delivery to first participant enrollment) as compared by trial year and type of IRB used. The median start-up time for the top 10% of sites was also reported. Secondary outcomes included time to site regulatory approval, time to contract execution, and time to site activation.

**Results:**

For the 9 RCTs included, the median site start-up time shortened only slightly over time from 267 days (interquartile range [IQR], 185-358 days) for 2004-2007 trials to 237 days (IQR, 162-343 days) for 2008-2012 trials (overall median, 255 days [IQR, 177-350 days]; *P* < .001). For the top 10% of sites, median start-up time was 107 days (IQR, 95-121 days) for 2004-2007 trials vs 104 days (IQR, 84-118 days) for 2008-2012 trials (overall median, 106 days [IQR, 90-120 days]; *P* = .04). The median start-up time was shorter among sites using a central IRB (199 days [IQR, 140-292 days]) than those using a local IRB (287 days [IQR, 205-390 days]; *P* < .001).

**Conclusions and Relevance:**

This cohort study of North American research sites in large cardiovascular RCTs found a duration of nearly 9 months from the time of study protocol delivery to the first participant enrollment; this metric was only slightly shortened during the study period but was reduced to less than 4 months for top-performing sites. These findings suggest that the use of central IRBs has the potential to improve RCT efficiency.

## Key Points

**Question:**

What are the start-up times needed to reach various milestones for North American research sites in large cardiovascular trials, and how do these milestones vary by time and regulatory process?

**Findings:**

In this cohort study including data from 9 clinical trials, the median start-up time (from study protocol delivery to first participant enrollment) was 255 days, which significantly improved from 267 days for trials in 2004-2007 to 237 days for trials in 2008-2012. Sites using a central vs local regulatory process had a significantly reduced start-up time of 199 vs 287 days, respectively.

**Meaning:**

In addition to providing benchmark metrics, these data demonstrate modest improvement over time and suggest that use of central institutional review boards may enhance trial efficiency.

## Teaser

This cohort study uses data from 9 randomized clinical cardiovascular trials to assess the start-up times of various milestones for North American research sites and how these metrics vary by time and regulatory process.

## Introduction

Over the past 4 decades, large randomized clinical trials (RCTs) have been critical in advancing care for patients with cardiovascular disease; these RCTs remain the foundation for practicing evidence-based, clinical medicine.^1^ However, conducting such RCTs on a large scale necessitates the coordination of various stakeholders (research organizations, practitioners, patients, industry, and professional societies) and numerous sites enrolling high numbers of research participants. The burden of cost and the length of time required for these types of trials have previously been described as extensive and increasing.^2,3,4^

Despite the importance of RCTs, more than 90% of guideline recommendations made by the largest cardiovascular societies are not validated by such RCTs, leading some to call for new trial design methodologies.^1,5,6^ The inefficiencies in discovering practice-changing treatment options has led to the prominence of pragmatic clinical trials that facilitate enrollment of a diverse study population, inclusion of health care professionals in community settings, public-private partnerships, and patient-centered outcomes.^7,8,9,10^ Owing to the complexity of conducting large RCTs, several institutions across North America have developed academic research organizations to manage the logistics and challenges that may arise. The Duke Clinical Research Institute (DCRI) is one such organization that has coordinated several large cardiovascular RCTs over the past 3 decades.^11,12,13,14,15,16,17,18,19,20,21^

Though structural changes have been made to RCT designs, few studies quantitatively assess whether such modifications to design and regulatory processes improve trial efficiency. In particular, the time taken to enroll a site’s first participant after protocol finalization remains largely unknown but represents an opportunity for improvement. To quantitatively evaluate trial efficiency, a comprehensive assessment of the DCRI’s experience with 9 large cardiovascular clinical trials enrolling participants from 2004 to 2017 was undertaken.

## Methods

### Clinical Trials

Nine consecutive cardiovascular outcomes trials^20,22,23,24,25,26,27,28,29^ coordinated by the DCRI with available data were selected for this analysis. These trials—with the earliest trial beginning enrollment on July 13, 2004, and the latest trial ending enrollment on February 1, 2017—were managed by global collaborations between pharmaceutical companies and leading academic institutions. In addition, the trials covered a wide range of cardiovascular diagnoses inclusive of acute coronary syndrome, atrial fibrillation, heart failure, prevention, type 2 diabetes, and peripheral artery disease (Table 1). For consideration of inclusion, trials had to have completed enrollment with publication of results in their target journal. Although these trials were conducted globally, the present analysis reviewed those sites managed within North America. All trial protocols were approved by multiple parties, including the US Food and Drug Administration, pharmaceutical company committees, and large academic steering committees, before being sent to sites in North America. The DCRI was the primary or coprimary coordinating center for each of the trials, with the rights held by the DCRI and global academic leaders for analysis and publication of the clinical trial results. Operational metrics were collected for each trial for each DCRI-managed North American site. For analysis purposes, the included trials were stratified into 2 groups based on trial enrollment start year: early trials dated from July 13, 2004, to December 18, 2007 (Assessment of Pexelizumab in Acute Myocardial Infarction [APEX-AMI], Improved Reduction of Outcomes: Vytorin Efficacy International Trial [IMPROVE-IT], Rivaroxaban vs Warfarin in Nonvalvular Atrial Fibrillation [ROCKET-AF], Acute Study of Clinical Effectiveness of Nesiritide in Decompensated Heart Failure [ASCEND-HF], and Thrombin Receptor Antagonist for Clinical Event Reduction in Acute Coronary Syndrome [TRACER]),^20,22,23,24,25^ and later trials dated from December 1, 2008, to December 1, 2012 (Trial Evaluating Cardiovascular Outcomes with Sitagliptin [TECOS], Exenatide Study of Cardiovascular Event Lowering [EXSCEL], Evaluation of Cardiovascular Outcomes After an Acute Coronary Syndrome During Treatment With Alirocumab [ODYSSEY], and Examining Use of Ticagrelor in Peripheral Artery Disease [EUCLID]).^26,27,28,29^ This study was considered exempt from obtaining participant consent by the institutional review board (IRB) of Duke University due to the aggregation of operational data that did not involve specific participant data. This study adhered to the Strengthening the Reporting of Observational Studies in Epidemiology (STROBE) reporting guideline.

**Table 1.** Trial Attributes and No. of North American Sites by Milestone

Trial name	Year of enrollment start	Cardiovascular area of research	No. of sites to have reached the specific milestones (% frequency)		
			Regulatory approval	Contract execution	Enrollment of first participant
APEX-AMI^a^	Q2, 2004	ACS/inpatient	207 (9.3)	161 (7.7)	164 (9.0)
IMPROVE-IT	Q3, 2005	ACS/inpatient & outpatient	252 (11.3)	248 (11.9)	201 (11.0)
ROCKET-AF	Q3, 2006	AF/outpatient	283 (12.7)	273 (13.0)	203 (11.1)
ASCEND-HF	Q1, 2007	Acute HF/inpatient	280 (12.6)	275 (13.1)	213 (11.6)
TRACER	Q3, 2007	ACS/inpatient	286 (12.9)	222 (10.6)	248 (13.5)
TECOS	Q3, 2008	CV prevention/outpatient	210 (9.4)	205 (9.8)	187 (10.2)
EXSCEL	Q1, 2010	CV prevention/outpatient	207 (9.3)	207 (9.9)	188 (10.3)
ODYSSEY	Q3, 2012	CV prevention/outpatient	330 (14.8)	333 (15.9)	286 (15.6)
EUCLID	Q3, 2012	PAD/outpatient	170 (7.6)	168 (8.0)	142 (7.8)

Abbreviations: ACS, acute coronary syndrome; AF, atrial fibrillation; CV, cardiovascular; HF, heart failure; PAD, peripheral artery disease; Q, quarter.

^a^ Trial name expansions are as follows: APEX-AMI, Assessment of Pexelizumab in Acute Myocardial Infarction; ASCEND-HF, Acute Study of Clinical Effectiveness of Nesiritide in Decompensated Heart Failure; EUCLID, Examining Use of Ticagrelor in Peripheral Artery Disease; EXSCEL, Exenatide Study of Cardiovascular Event Lowering; IMPROVE-IT, Improved Reduction of Outcomes: Vytorin Efficacy International Trial; ODYSSEY, Evaluation of Cardiovascular Outcomes After an Acute Coronary Syndrome During Treatment With Alirocumab; ROCKET-AF, Rivaroxaban vs Warfarin in Nonvalvular Atrial Fibrillation; TECOS, Trial Evaluating Cardiovascular Outcomes with Sitagliptin; TRACER, Thrombin Receptor Antagonist for Clinical Event Reduction in Acute Coronary Syndrome.

### Operational Metrics and Definitions

**Figure 1.**
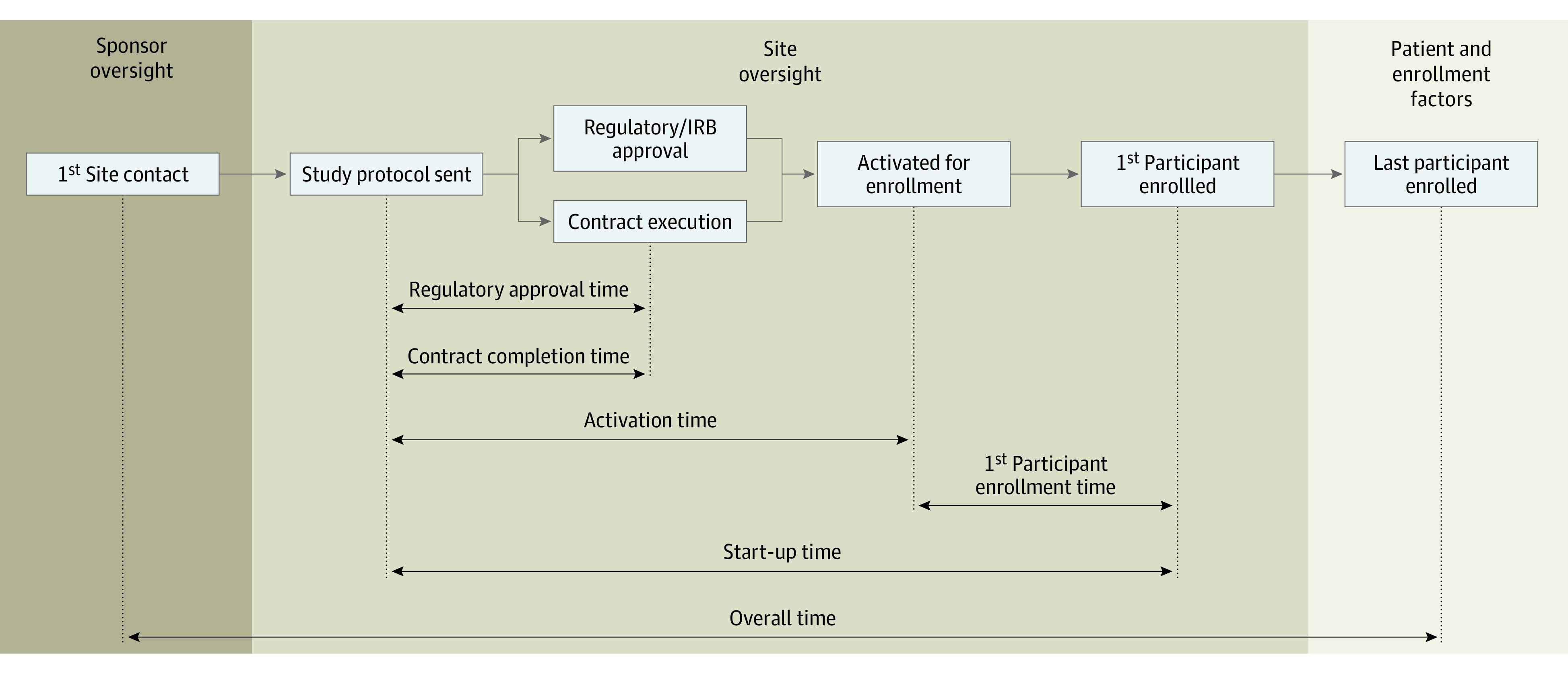
Time Intervals for Various Trial Milestones The current study addresses the “site oversight” portion of the trial approval process. In the schematic, the regulatory approval and the contract execution processes occur concurrently and thus may have different end times. IRB indicates institutional review board.

Numerous factors comprise the efficiency of overall time between site contact and enrollment conclusion. The current study examined several key operational start-up metrics within the purview of an individual site that achieved each milestone; these metrics were routinely and systematically collected for each site managed by the DCRI during trial start-up. These metrics comprised the start-up time, defined as the time from when the final study protocol was sent to the site to when the first participant was enrolled. As outlined graphically in Figure 1, in addition to start-up time, 3 milestones were monitored, including time from when the protocol was sent to the site to each of the following: (1) regulatory approval at the site (defined as the site having obtained ethics committee approval and having completed the necessary regulatory documents for study drug shipment), (2) contract execution, and (3) activation (defined as the site having authorization to enroll, which incorporates both regulatory approval and contract execution). Furthermore, the time from activation to first participant enrollment was collected. Finally, the time for regulatory approval was stratified based on the use of a central vs local IRB process.

### Statistical Analysis

Categorical variables were presented as counts with percentages, and continuous variables were reported as medians with interquartile ranges. Cumulative distribution function plots were used to summarize the distribution of the time to each milestone for each individual trial and for early vs contemporary trials. Box plots were generated showing the time to reach either regulatory approval or contract execution for each individual trial and for early vs contemporary trials. The Wilcoxon rank sum procedure was used to test for a difference in the median time to each milestone across the 2 temporarily stratified trial groupings. The χ^2^ test was used to assess the difference in the proportion of sites that had contract execution before regulatory approval between early and contemporary trials. Time was measured in number of days. Unless otherwise noted, all hypothesis tests were 2-sided, and *P* < .05 was interpreted as statistically significant. Analyses were interpreted as exploratory, and thus no formal adjustments for multiple hypothesis testing were made. Missing values were excluded from descriptive statistical summaries. All analyses were performed from December 4, 2019, to January 11, 2021, using SAS, version 9.4 (SAS Institute Inc).

## Results

**Figure 2.**
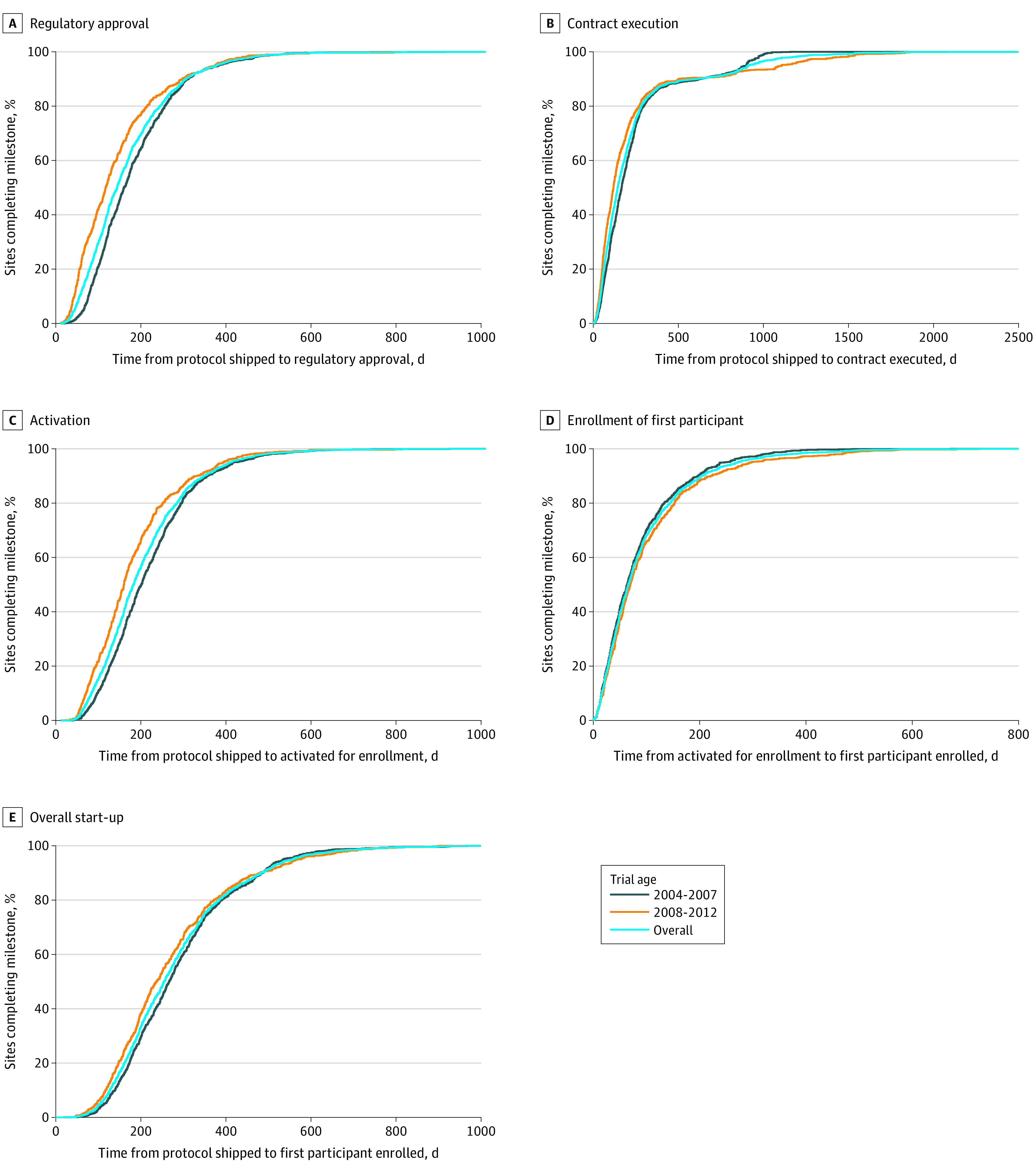
Time to Milestone by 2004-2007 vs 2008-2012 Trials The time to various milestones (A, regulatory approval; B, contract execution; C, activation; D, enrollment of first participant; and E, overall start-up) are stratified by early trials from 2004-2007 (Assessment of Pexelizumab in Acute Myocardial Infarction [APEX-AMI], Improved Reduction of Outcomes: Vytorin Efficacy International Trial [IMPROVE-IT], Rivaroxaban vs Warfarin in Nonvalvular Atrial Fibrillation [ROCKET-AF], Acute Study of Clinical Effectiveness of Nesiritide in Decompensated Heart Failure [ASCEND-AF], and Thrombin Receptor Antagonist for Clinical Event Reduction in Acute Coronary Syndrome [TRACER]) and contemporary trials from 2008-2012 (Trial Evaluating Cardiovascular Outcomes with Sitagliptin [TECOS], Exenatide Study of Cardiovascular Event Lowering [EXSCEL], Evaluation of Cardiovascular Outcomes After an Acute Coronary Syndrome During Treatment With Alirocumab [ODYSSEY], and Examining Use of Ticagrelor in Peripheral Artery Disease [EUCLID]).

The start of enrollment across the 9 selected trials^20,22,23,24,25,26,27,28,29^ ranged from July 13, 2004, to December 1, 2012 (Table 1). The times to various trial start-up milestones are described in Table 2 for all sites and for the top 10% of sites for each trial for each milestone. The median overall start-up time was 255 days (IQR, 177-350 days), which improved to 237 days (IQR, 162-343 days) for 2008-2012 trials compared with 267 days (IQR, 185-358 days) for 2004-2007 trials (*P* < .001). For the top 10% of sites, median start-up time was 107 days (IQR, 95-121 days) for 2004-2007 trials to 104 days (IQR, 84-118 days) for 2008-2012 trials (overall median, 106 days [IQR, 90-120 days]; *P* = .04). The median time to complete regulatory approval for all sites across all trials was 132 days (IQR, 78-209 days). This process improved to 105 days (IQR, 51-177 days) for 2008-2012 trials compared with 149 days (IQR, 97-224 days) for 2004-2007 trials (*P* < .001). Similarly, the median time to contract execution was 143 days (IQR, 74-250 days) overall and improved to 119 days (IQR, 59-223 days) for 2008-2012 trials compared with 167 days (IQR, 92-262 days) for 2004-2007 trials (*P* < .001). Median time to activation was 171 days (IQR, 114-246 days) and was followed by an additional 66 days (IQR, 33-124 days) to first participant enrollment. These milestones improved to 149 days (IQR, 97-217 days) and 70 days (IQR, 37-134 days), respectively, for 2008-2012 trials (*P* < .001) compared with 189 days (IQR, 129-265 days) and 64 days (IQR, 32-117 days), respectively, for 2004-2007 trials (*P* = .01). Comparable data for the top 10% of enrolling sites can be found in Table 2. The cumulative distribution function plots for 2004-2007 and 2008-2012 trials for each milestone are highlighted in Figure 2 (individual trial cumulative distribution function plots can be found in eFigures 1-5 in the Supplement).

**Table 2.** Time to Trial Start-up Milestones, at All Sites and the Top 10% of Sites^a^

Milestone	Median (IQR) time to milestone, d							
	All sites				Top 10% of sites			
	All	2004-2007^b^	2008-2012^c^	*P* value^d^	All	2004-2007^b^	2008-2012^c^	*P* value^d^
Regulatory approval	132 (78-209)	149 (97-224)	105 (51-177)	<.001	34 (24-40)	36 (28-42)	32 (24-39)	<.001
Contract execution	143 (74-250)	167 (92-262)	119 (59-223)	<.001	31 (22-38)	30 (23-39)	31 (20-38)	<.001
Activation	171 (114-246)	189 (129-265)	149 (97-217)	<.001	56 (48-64)	59 (50-67)	55 (44-64)	<.001
Enrollment of first participant	66 (33-124)	64 (32-117)	70 (37-134)	.01	12 (7-14)	12 (8-15)	10 (7-14)	<.001
Overall start-up	255 (177-350)	267 (185-358)	237 (162-343)	<.001	106 (90-120)	107 (95-121)	104 (84-118)	.04

^a^ Trial name expansions are as follows: APEX-AMI, Assessment of Pexelizumab in Acute Myocardial Infarction; ASCEND-HF, Acute Study of Clinical Effectiveness of Nesiritide in Decompensated Heart Failure; EUCLID, Examining Use of Ticagrelor in Peripheral Artery Disease; EXSCEL, Exenatide Study of Cardiovascular Event Lowering; IMPROVE-IT, Improved Reduction of Outcomes: Vytorin Efficacy International Trial; ODYSSEY, Evaluation of Cardiovascular Outcomes After an Acute Coronary Syndrome During Treatment With Alirocumab; ROCKET-AF, Rivaroxaban vs Warfarin in Nonvalvular Atrial Fibrillation; TECOS, Trial Evaluating Cardiovascular Outcomes with Sitagliptin; TRACER, Thrombin Receptor Antagonist for Clinical Event Reduction in Acute Coronary Syndrome.

^b^ The 2004-2007 trials include APEX-AMI, IMPROVE-IT, ROCKET-AF, ASCEND-AF, and TRACER.

^c^ The 2008-2012 trials include TECOS, EXSCEL, ODYSSEY, and EUCLID.

^d^ The *P* value describes difference between the 2 time intervals. Wilcoxon rank sum tests are used to analyze continuous variables.

To explore whether contract execution or regulatory document completion was associated with the pacing of start-up times, the median times for contract execution and regulatory approval are shown, depending on which activity was first completed, in eTable 1 in the Supplement. For sites completing regulatory approval first, an additional 46 days (IQR, 13-155 days) were needed for contract execution. For sites with contract execution first, an additional 30 days (IQR, 12-63 days) were needed for regulatory approval. These times were slightly faster for both metrics for the 2008-2012 trials compared with the 2004-2007 trials (regulatory approval first: 2008-2012 trials, 33 days [IQR, 8-111 days] vs 2004-2007 trials, 60 days [IQR, 20-210 days]; *P* < .001; and contract execution first: 2008-2012 trials, 27 days [IQR, 8-61 days] vs 2004-2007 trials, 33 days [IQR, 14-63 days]; *P* = .03). Individual trial data can be found in eTable 2 in the Supplement. Additionally, the number of sites completing their contracts before obtaining regulatory approval was 1104 (49.6%) for all sites, which was fewer at 427 (46.6%) for 2008-2012 trials compared with 680 (52.0%) for 2004-2007 trials (*P* = .02). The box plot distributions of days to regulatory approval and contract execution are shown in eFigure 6, with individual trials shown in eFigure 7 in the Supplement.

To compare whether the type of IRB regulatory approval process was associated with the time to IRB regulatory approval or overall start-up, the use of a central or local IRB process was compared across IRB type and across 2004-2007 vs 2008-2012 trials (Table 3). Individual trial data can be found in eTables 3 and 4 in the Supplement.

**Table 3.** Time to Milestone by IRB Type

Trial^a^ grouping	IRB type	Regulatory approval			Overall start-up		
		No. of sites, (%)^b^	Time to regulatory approval, median (25th-75th percentile), d	*P* value^c^	No. of sites, (%)^d^	Time to overall start-up, median (25th-75th percentile), d	*P* value^c^
All trials	Central	798 (35.9)	78 (45-124)	<.001	681 (37.5)	199 (140-292)	<.001
	Local	1209 (54.3)	165 (112-244)		1005 (55.5)	287 (205-390)	
2004-2007 Trials^e^	Central	258 (19.7)	92 (64-146)	<.001	208 (20.5)	203 (139-304)	<.001
	Local	863 (66.0)	161 (110-237)		697 (68.7)	278 (200-375)	
2008-2012 Trials^f^	Central	540 (58.9)	71 (41-112)	<.001	473 (59.3)	196 (141-288)	<.001
	Local	346 (37.7)	181 (121-274)		308 (38.6)	305 (222-422)	

Abbreviation: IRB, institutional review board.

^a^ Trial name expansions are as follows: APEX-AMI, Assessment of Pexelizumab in Acute Myocardial Infarction; ASCEND-HF, Acute Study of Clinical Effectiveness of Nesiritide in Decompensated Heart Failure; EUCLID, Examining Use of Ticagrelor in Peripheral Artery Disease; EXSCEL, Exenatide Study of Cardiovascular Event Lowering; IMPROVE-IT, Improved Reduction of Outcomes: Vytorin Efficacy International Trial; ODYSSEY, Evaluation of Cardiovascular Outcomes After an Acute Coronary Syndrome During Treatment With Alirocumab; ROCKET-AF, Rivaroxaban vs Warfarin in Nonvalvular Atrial Fibrillation; TECOS, Trial Evaluating Cardiovascular Outcomes with Sitagliptin; TRACER, Thrombin Receptor Antagonist for Clinical Event Reduction in Acute Coronary Syndrome.

^b^ Across all trials, 218 sites (9.8%) did not have type of institutional review board specified.

^c^ The *P* value describes the difference in time to milestone between central and local institutional review board processes.

^d^ Across all trials, 126 sites (7.0%) did not have type of institutional review board specified.

^e^ The 2004-2007 trials include APEX-AMI, IMPROVE-IT, ROCKET-AF, ASCEND-AF, and TRACER.

^f^ The 2008-2012 trials include TECOS, EXSCEL, ODYSSEY, and EUCLID.

For the regulatory analysis, 2225 sites were included in the analysis, with 798 sites (35.9%) using a central IRB, 1209 sites (54.3%) using a local IRB, and 218 sites (9.8%) without information specifying the type of IRB used. The median time to regulatory approval in sites using a central IRB was 78 days (IQR, 45-124 days), which was more than half the time seen in sites using a local IRB (165 days [IQR, 112-244 days]; *P* < .001). The time to regulatory approval was faster for sites using a central IRB regardless of whether they were 2004-2007 or 2008-2012 trials (2004-2007 trials with central IRB, 92 days [IQR, 64-146 days] vs local IRB, 161 days [IQR, 110-237 days]; *P* < .001; and 2008-2012: central IRB, 71 days [IQR, 41-112 days] vs local IRB, 181 days [IQR, 121-274 days]; *P* < .001), and contemporary trials more often used a central IRB process (540 sites [58.9%] vs 258 sites [19.7%]). The median time to overall start-up was also significantly less for sites using a central vs local IRB (199 days [IQR, 140-292 days] vs 287 days [IQR, 205-390 days]; *P* < .001).

## Discussion

In this study of 9 large cardiovascular RCTs^20,22,23,24,25,26,27,28,29^ conducted at the DCRI, we found that the overall site start-up time in North America was nearly 9 months, although top-performing sites for each trial completed their start-up in less than 4 months. There was a significant but modest improvement in this metric when comparing early to contemporary trials, possibly related to the outpatient setting in which these later trials were conducted. Contemporary trials more frequently used a central IRB and had faster times to regulatory approval when compared with earlier trials and sites using a local IRB. To our knowledge, this study for the first time quantitatively characterizes these performance metrics and provides insight into target areas for improvement.

With increasing global burden of cardiovascular disease, RCTs remain the foundation for answering challenging clinical questions and investigating new therapies. Novel approaches are needed to improve trial efficiency while maintaining rigorous oversight, ensuring protection of human participants, and keeping the interests of industry and academia transparent. The balance of various stakeholders with diverse interests is evidenced by the 255-day median start-up time observed in our sample. Krafcik et al^30^ described a 319-day median start-up time in a review of 38 heterogenous studies ranging from 2004 to 2016 in Boston, Massachusetts. Similarly, a 174-day start-up time across 13 Saudi clinical trials was reported by Abu-Shaheen et al,^31^ with a significant portion of that time dedicated to obtaining local IRB approval. To limit loss of resources spent on the contractual process without regulatory approval and vice versa, many institutions complete the regulatory approval process and contract negotiations consecutively rather than concurrently. In addition, numerous delays can be encountered. For example, sites are frequently approached to participate in multiple trials and must determine which to conduct at their institutions; protocols may change before activation and require additional review; and centers with large portfolios often have internal review committees that deliberate over how to allocate resources and select patient populations for new projects. Not addressed in the current study is the financial and human capital required for site training, which has taken on an even greater burden in recent years with the use of centralized systems to manage and track individual training.

In agreement with previously published data,^30^ our data suggest improved overall efficiency with the use of central IRBs, and the rationale for multiple sites reviewing clinical trial protocols through individual IRBs should be reconsidered. Such multicenter clinical trial protocols are often reviewed by several independent parties, including academic leaders, data monitoring committees, and regulatory agencies. As promoted by the National Institutes of Health, the use of a single IRB system that streamlines local IRB reviews for approved protocols^32^ can be a significant step toward enhancing regulatory efficiency while preserving ethical principles and participant protections.

Contract negotiations and budget development have also become more complex, with common key areas of contract disagreement including terms of confidentiality, rights to data and publication, and intellectual property. Often, these negotiations occur repeatedly with subsequent protocols with the same pharmaceutical sponsor and academic group, which can cause delay. Such delays can be improved by using master service agreements and proactively establishing alternate language and form commitments for sponsors and sites during contract negotiations to reduce turnaround times on proposed changes.

Though our data suggest modest improvement in trial start-up efficiency metrics over the past 15 years, more effort is needed to expedite this process while preserving individual stakeholder interests. All key parties in clinical research, including pharmaceutical companies, clinical researchers, participants, and regulatory agencies, have a vested interest in optimization, but there is often conflict and inertia that continue to make progress challenging. Pharmaceutical companies must ensure that trials can meet regulatory scrutiny while protecting shareholder value and pharmaceutical portfolios; academic institutions need specific research rights to protect institutional integrity and tax-exempt status, which requires rights to trial data sets and publication of results; and regulatory agencies are focused on drug safety and patient advocacy. Multiple collaborative approaches have ensued, including collegial forums, such as the Clinical Trial Transformation Initiative formed in 2007 by the Center for Drug Evaluation and Research.^33^ Broader use of master service agreements, streamlined protocol templates, and efforts such as the DCRI’s Rapid Start Up initiative, which uses prenegotiated, rapidly replicated contract language for key areas, can all serve to reduce delays and improve efficiency. Furthermore, although there is interest in globalization of clinical research by exporting research efforts to areas with fewer cost, regulation, and contractual barriers, there should be an equal effort in validating and enhancing North American trial operations metrics.^34^

Our data from the top 10% of sites consistently show that sites can proceed through the site initiation procedures efficiently. However, additional work is needed to evaluate the specific measures that these sites take to achieve their level of productivity beyond the use of central IRBs. Large RCTs should continue to track and report their site metrics to provide objective data to promote an evidence-based operational plan and support trial design changes. Recent scientific efforts to curb the public health effects of the SARS-CoV-2 pandemic demonstrate the ability of large, complex systems to work efficiently across the sometimes diverging interests of various global stakeholders. The epidemic of cardiovascular disease is arguably of equal imminent importance. Although tremendous public financial and human resources are dedicated to improving its morbidity and mortality, a concerted and efficient system for implementing these resources has yet to be developed. In the coming years, clinical and regulatory communities will continue to require outcomes studies to identify beneficial therapies and drive integration of these interventions into clinical guidelines and practice. These data provide insight into how RCT design and development can be optimized to efficiently and effectively deliver the results needed for practicing evidence-based, clinical medicine.

### Limitations

This study had several limitations. The included trials were inherently heterogenous with regard to enrollment environment (acute inpatient vs outpatient), time of follow-up, and area of cardiovascular research, which may have limited the strength of the observed comparisons. This limitation is mitigated some in that all trials were coordinated through a single academic research organization. Further, there were no direct comparisons for start-up metrics between sites that were managed by contract research organizations vs those managed by the sponsor or various site-specific features (eg, academic vs nonacademic or population density). The current study was also unable to account for the inherent delay associated with intermittent meetings of local IRB review committees, which disproportionately affects sites using this regulatory process. Additionally, sites that received protocols and contracts but did not enroll any patients represent significant cost and resource usage but were not captured in this study. Finally, given the publication delay, we were unable to include more contemporary and ongoing trials in the current study.

## Conclusions

In this cohort study, research sites in North America that participated in large cardiovascular RCTs required nearly 9 months from time of initial contact to first participant enrollment, although these measures have modestly improved over time. The use of central IRBs may enhance RCT start-up efficiency, but more work is needed to ensure the timely implementation of a research protocol while protecting the interests of various stakeholders.

## Notes

Supplement. **eFigure 1.** Regulatory Approval Time by Trial **eFigure 2.** Contract Execution Time by Trial **eFigure 3.** Activation Time by Trial **eFigure 4.** Enrollment of First Participant Time by Trial **eFigure 5.** Overall Start-Up Time by Trial **eFigure 6.** Side-by-Side Boxplots of Regulatory Approval and Contract Execution by 2004-2007 vs 2008-2012 Trials **eFigure 7.** Side-by-Side Boxplots of Regulatory Approval and Contract Execution by Trial **eTable 1.** Milestone Pacing Site Activation **eTable 2.** Milestone Pacing to Site Activation for Individual Trials **eTable 3.** Time to Regulatory Approval for Individual Trials by IRB Type **eTable 4.** Time to Overall Start-Up for Individual Trials by IRB Type
