# Supplementary material for: Assessment of North American Clinical Research Site Performance During the Start-up of Large Cardiovascular Clinical Trials
Source: JAMA Netw Open. 2021 Jul 23;4(7):e2117963. doi: 10.1001/jamanetworkopen.2021.17963 (PMC9435961; doi:10.1001/jamanetworkopen.2021.17963)
Supplement: Supplement. — eFigure 1. Regulatory Approval Time by Trial eFigure 2. Contract Execution Time by Trial eFigure 3. Activation Time by Trial eFigure 4. Enrollment of First Participant Time by Trial eFigure 5. Overall Start-Up Time by Trial eFigure 6. Side-by-Side Boxplots of Regulatory Approval and Contract Execution by 2004-2007 vs 2008-2012 Trials eFigure 7. Side-by-Side Boxplots of Regulatory Approval and Contract Execution by Trial eTable 1. Milestone Pacing Site Activation eTable 2. Milestone Pacing to Site Activation for Individual Trials eTable 3. Time to Regulatory Approval for Individual Trials by IRB Type eTable 4. Time to Overall Start-Up for Individual Trials by IRB Type [file jamanetwopen-e2117963-s001.pdf]

Supplementary Online Content

Goyal A, Schibler T, Alhanti B, et al. Assessment of North American clinical research site performance during the start-up of large cardiovascular clinical trials. *JAMA Netw Open*. 2021;4(7):e2117963. doi:10.1001/jamanetworkopen.2021.17963

eFigure 1. Regulatory Approval Time by Trial

eFigure 2. Contract Execution Time by Trial

eFigure 3. Activation Time by Trial

eFigure 4. Enrollment of First Participant Time by Trial

eFigure 5. Overall Start-Up Time by Trial

eFigure 6. Side-by-Side Boxplots of Regulatory Approval and Contract Execution by 2004-2007 vs 2008-2012 Trials

eFigure 7. Side-by-Side Boxplots of Regulatory Approval and Contract Execution by Trial

eTable 1. Milestone Pacing Site Activation

eTable 2. Milestone Pacing to Site Activation for Individual Trials

eTable 3. Time to Regulatory Approval for Individual Trials by IRB Type

eTable 4. Time to Overall Start-Up for Individual Trials by IRB Type

This supplementary material has been provided by the authors to give readers additional information about their work.

© 2021 Goyal A et al. *JAMA Network Open.*

eFigure 1. Regulatory Approval Time by Trial

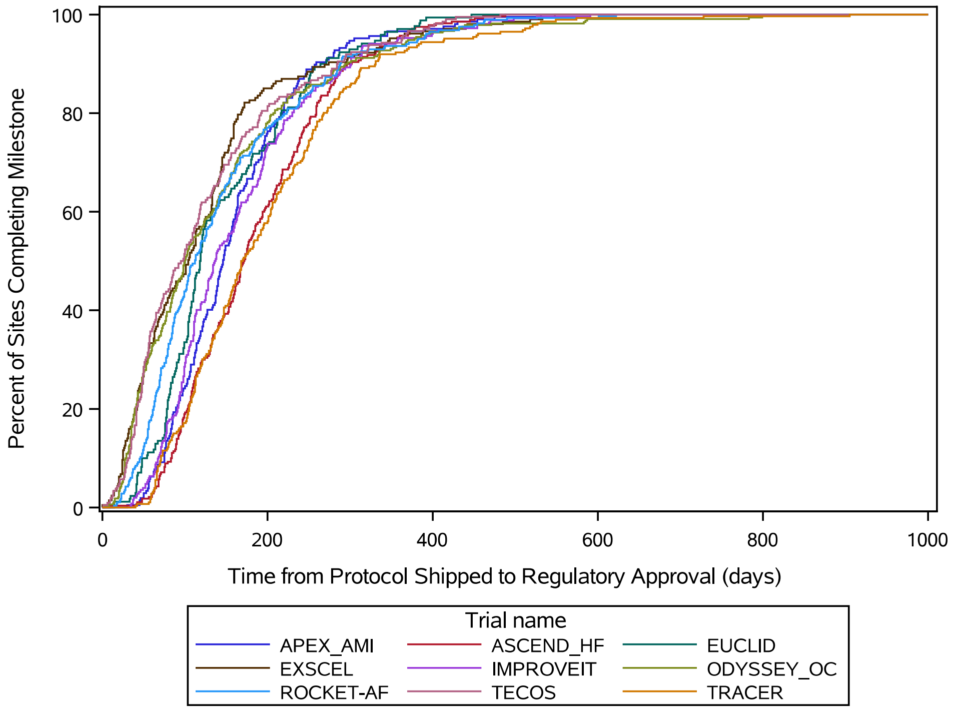

Caption: The time to regulatory approval for each trial represented by the percent of sites completing the milestone at a given time in days.

© 2021 Goyal A et al. *JAMA Network Open.*

eFigure 2. Contract Execution Time by Trial

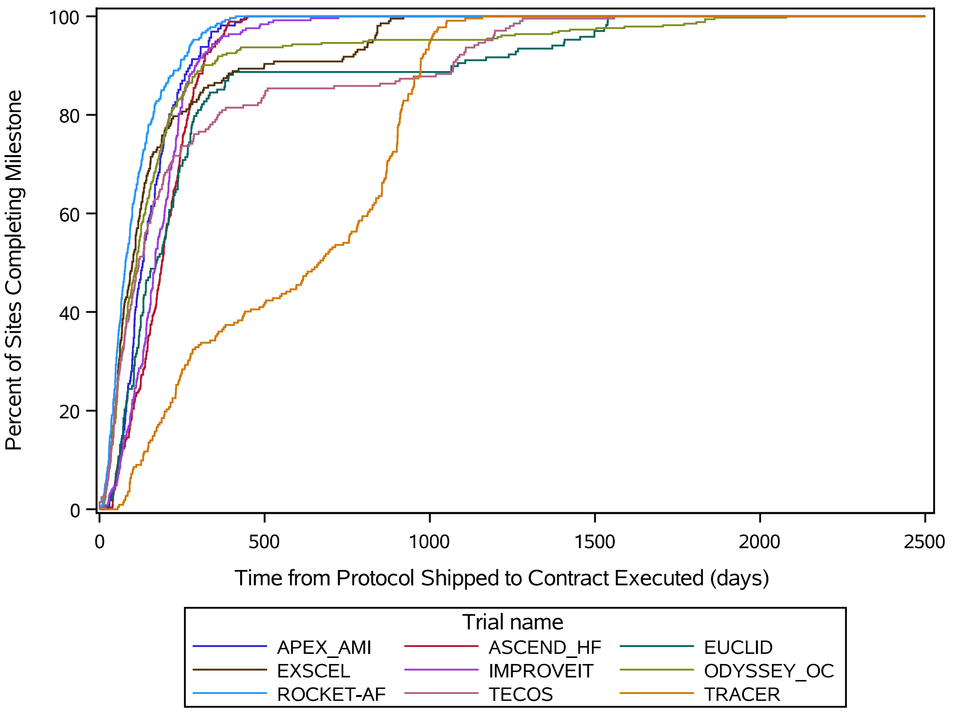

Caption: The time to contract execution for each trial represented by the percent of sites completing the milestone at a given time in days.

© 2021 Goyal A et al. *JAMA Network Open.*

eFigure 3. Activation Time by Trial

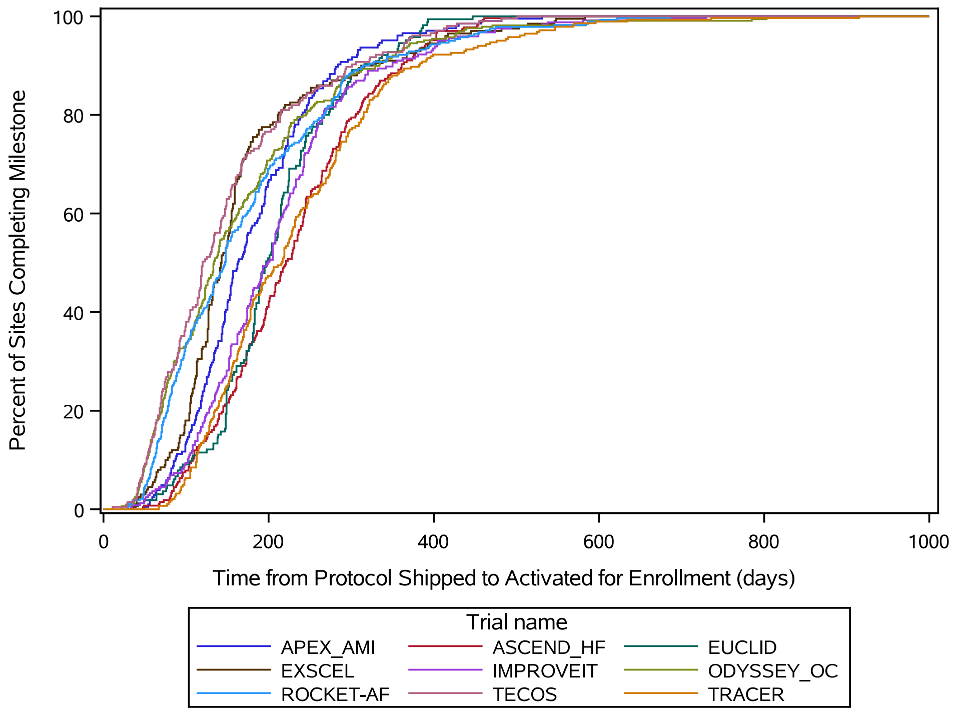

Caption: The activation time for each trial represented by the percent of sites completing the milestone at a given time in days.

© 2021 Goyal A et al. *JAMA Network Open.*

eFigure 4. Enrollment of First Participant Time by Trial

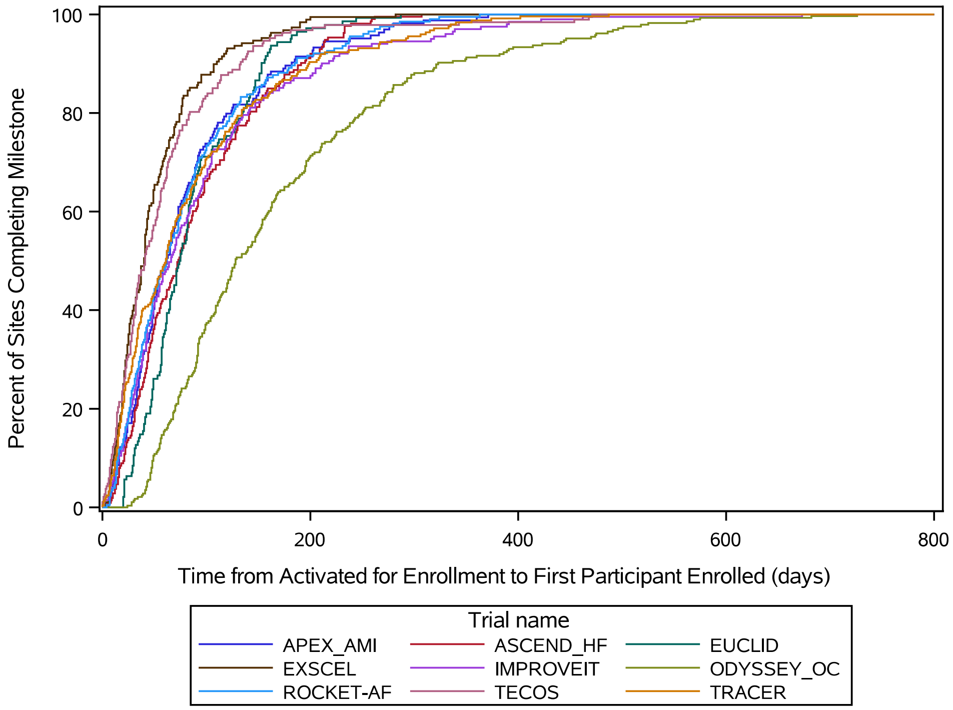

Caption: The time to enrollment of the first participant for each trial represented by the percent of sites completing the milestone at a given time in days.

© 2021 Goyal A et al. *JAMA Network Open.*

eFigure 5. Overall Start-Up Time by Trial

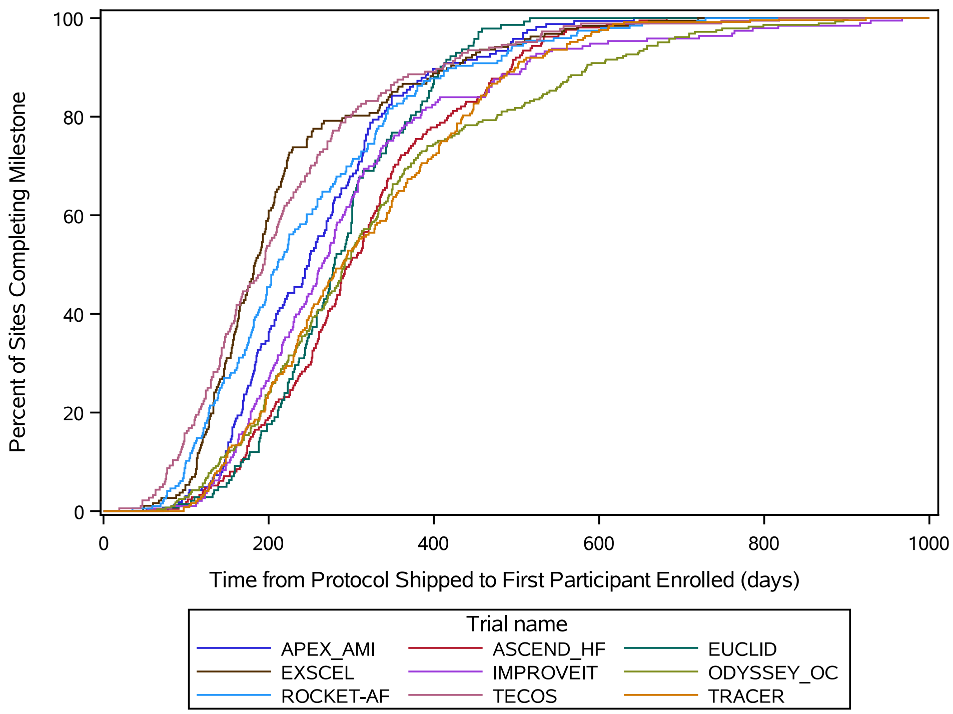

Caption: The overall start-up time for each trial represented by the percent of sites completing the milestone at a given time in days.

© 2021 Goyal A et al. *JAMA Network Open.*

eFigure 6. Side-by-Side Boxplots of Regulatory Approval and Contract Execution by 2004-2007 vs 2008-2012 Trials

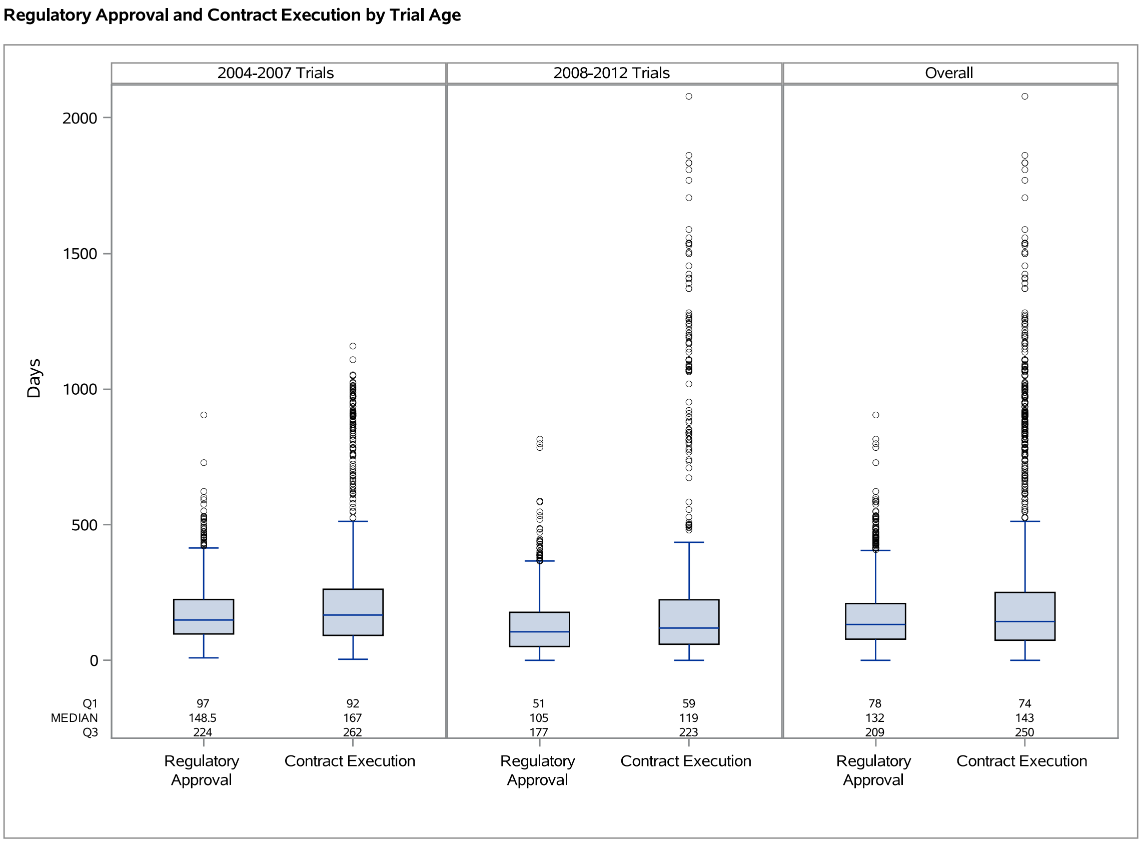

Caption: The boxplots show the days required to reach both regulatory approval and contract execution stratified by early trials from 2004-2007 (APEX-AMI, IMPROVE-IT, ROCKET-AF, ASCEND-AF, & TRACER) and contemporary trials from 2008-2012 (TECOS, EXSCEL, ODYSSEY, & EUCLID).

© 2021 Goyal A et al. *JAMA Network Open.*

eFigure 7. Side-by-Side Boxplots of Regulatory Approval and Contract Execution by Trial

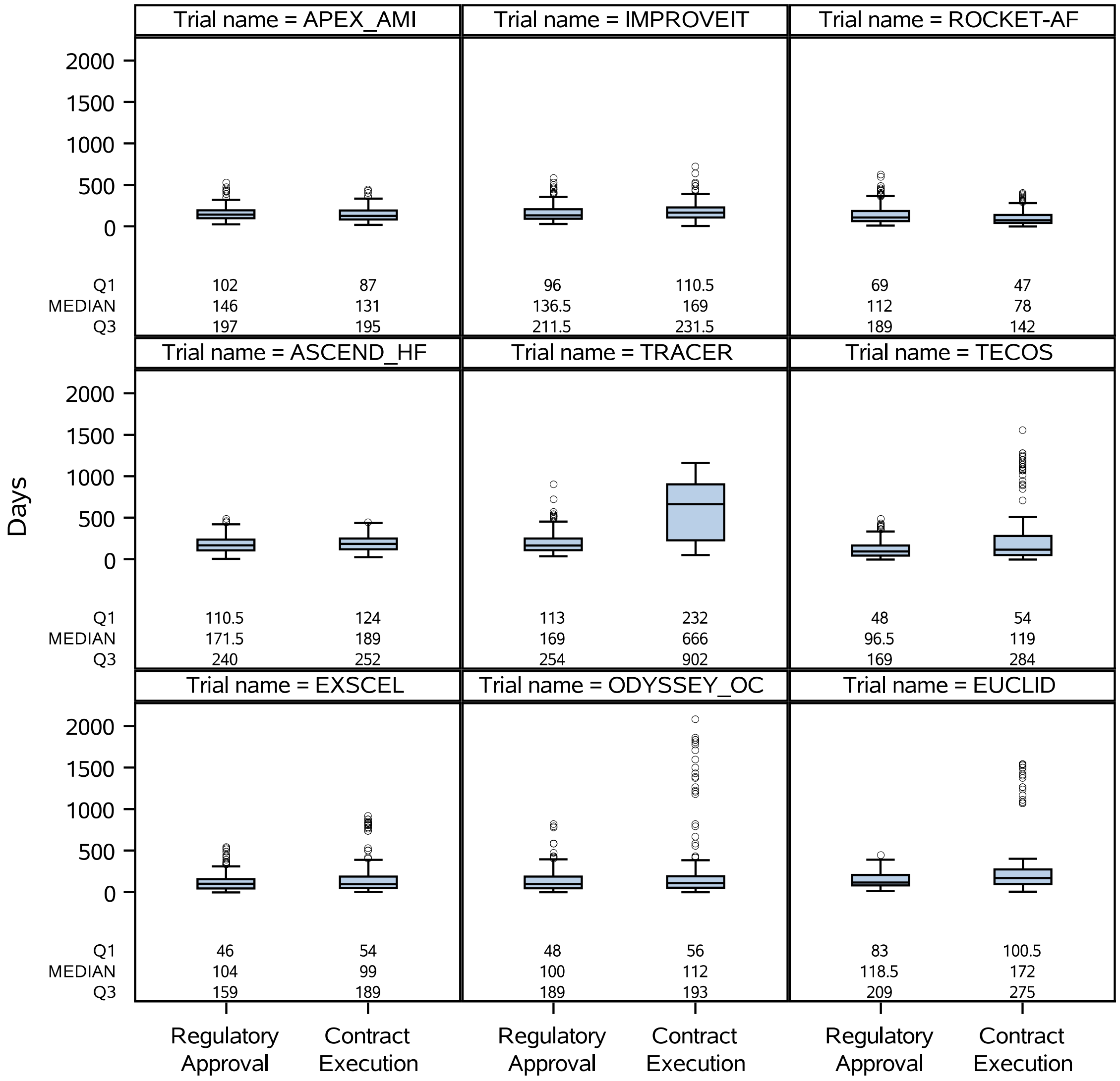

Trial	Measure	Regulatory Approval	Contract Execution
APEX_AMI	Q1	102	87
APEX_AMI	MEDIAN	146	131
APEX_AMI	Q3	197	195
IMPROVEIT	Q1	96	110.5
IMPROVEIT	MEDIAN	136.5	169
IMPROVEIT	Q3	211.5	231.5
ROCKET-AF	Q1	69	47
ROCKET-AF	MEDIAN	112	78
ROCKET-AF	Q3	189	142
ASCEND_HF	Q1	110.5	124
ASCEND_HF	MEDIAN	171.5	189
ASCEND_HF	Q3	240	252
TRACER	Q1	113	232
TRACER	MEDIAN	169	666
TRACER	Q3	254	902
TECOS	Q1	48	54
TECOS	MEDIAN	96.5	119
TECOS	Q3	169	284
EXSCEL	Q1	46	54
EXSCEL	MEDIAN	104	99
EXSCEL	Q3	159	189
ODYSSEY_OC	Q1	48	56
ODYSSEY_OC	MEDIAN	100	112
ODYSSEY_OC	Q3	189	193
EUCLID	Q1	83	100.5
EUCLID	MEDIAN	118.5	172
EUCLID	Q3	209	275

Caption: The boxplots show the days required to reach both regulatory approval and contract execution stratified by individual trial.

© 2021 Goyal A et al. *JAMA Network Open.*

eTable 1. Milestone Pacing Site Activation

Milestone	All Trials	2004-2007* Trials	2008-2012† Trials	P-value‡
Contract Complete First, (% of sites)	49.6%	52.0%	46.6%	0.02
Regulatory Approval first; Subsequent Time to Contract Execution (days)	46 (13, 155)	60 (20, 210)	33 (8, 111)	<0.001
Contract Execution first; Subsequent Time to Regulatory Approval (days)	30 (12, 63)	33 (14, 63)	27 (8, 61)	0.03

Continuous variables are described using median (25th, 75th percentile). Categorical variables are described using frequency (%). *2004-2007 trials: APEX-AMI, IMPROVE-IT, ROCKET-AF, ASCEND-AF, & TRACER; †2008-2012 trials: TECOS, EXSCEL, ODYSSEY, & EUCLID; ‡P-value describes difference between 2004-2007 and 2008-2012 trials.

eTable 2. Milestone Pacing to Site Activation for Individual Trials

Trial	Contract Execution First, (% of sites)	Regulatory Approval First; Subsequent Time to Contract Execution (days)	Contract Execution First; Subsequent Time to Regulatory Approval (days)
APEX-AMI	55.6%	20 (6, 46)	26 (7, 47)
IMPROVE-IT	45.1%	51 (18, 83)	28 (12, 51)
ROCKET-AF	76.3%	29 (8, 63)	44 (20, 74)
ASCEND-HF	55.2%	48 (18, 100)	29 (13, 58)
TRACER	25.2%	642 (377, 758)	40 (15, 84)
TECOS	47.5%	35 (6, 777)	24 (8, 54)
EXSCEL	49.5%	34 (5, 99)	30 (9, 71)
ODYSSEY	48.0%	22 (7, 69)	29 (9, 57)
EUCLID	39.5%	54 (23, 150)	25 (8, 55)

Continuous variables are described using median (25th, 75th percentile). Categorical variables are described using frequency (%).

© 2021 Goyal A et al. *JAMA Network Open.*

eTable 3. Time to Regulatory Approval for Individual Trials by IRB Type

Trial	IRB Type	Number of Sites, n	Time to Regulatory Approval, days
APEX-AMI	All sites	207	146 (102, 197)
	Local	207 (100%)	146 (102, 197)
IMPROVE-IT	All sites	252	137 (96, 212)
	Local	252 (100%)	137 (96, 212)
ROCKET-AF	All sites	283	112 (69, 189)
	Central	185 (65.4%)	84 (56, 130)
	Local	89 (31.4%)	194 (140, 286)
	Missing	9 (3.2%)	285 (156, 407)
ASCEND_HF	All sites	280	172 (111, 240)
	Central	15 (5.4%)	172 (109, 265)
	Local	90 (32.1%)	176 (127, 262)
	Missing	175 (62.5%)	167 (107, 236)
TRACER	All sites	286	169 (113, 254)
	Central	58 (20.3%)	134 (71, 216)
	Local	225 (78.6%)	183 (121, 258)
	Missing	3 (1.0%)	192 (90, 301)
TECOS	All sites	210	97 (48, 169)
	Central	97 (46.2%)	55 (41, 86)
	Local	94 (44.8%)	159 (100, 261)
	Missing	19 (9.0%)	102 (26, 193)
EXSCEL	All sites	207	104 (46, 159)
	Central	145 (70.0%)	65 (38, 117)
	Local	50 (24.2%)	194 (145, 332)
	Missing	12 (5.8%)	132 (62, 220)
ODYSSEY	All sites	330	100 (48, 189)
	Central	193 (58.5%)	58 (35, 102)
	Local	137 (41.5%)	190 (130, 276)
EUCLID	All sites	170	119 (83, 209)
	Central	105 (61.8%)	102 (76, 124)
	Local	65 (38.2%)	209 (121, 252)

Continuous variables are described using median (25th, 75th percentile). Categorical variables are described using frequency (%). IRB = institutional review board.

© 2021 Goyal A et al. *JAMA Network Open.*

eTable 4. Time to Overall Start-Up for Individual Trials by IRB Type

Trial	IRB Type	Number of Sites, n	Time to Overall Start-up, days
APEX-AMI	All sites	165	249 (175, 316)
	Local	165 (100%)	249 (175, 316)
IMPROVE-IT	All sites	193	265 (194, 344)
	Local	193 (100%)	265 (194, 344)
ROCKET-AF	All sites	196	211 (142, 328)
	Central	141 (71.9%)	181 (125, 246)
	Local	54 (27.6%)	332 (253, 412)
	Missing	1 (0.5%)	602 (602, 602)
ASCEND_HF	All sites	212	298 (230, 378)
	Central	15 (7.1%)	324 (268, 351)
	Local	89 (42.0%)	308 (226, 386)
	Missing	108 (50.9%)	289 (230, 380)
TRACER	All sites	248	290 (203, 413)
	Central	52 (21.0%)	258 (197, 370)
	Local	196 (79.0%)	299 (207, 423)
TECOS	All sites	184	194 (128, 273)
	Central IRB	78 (42.4%)	145 (98, 196)
	Local IRB	92 (50.0%)	232 (168, 345)
	Missing IRB	14 (7.6%)	205 (115, 262)
EXSCEL	All sites	187	183 (137, 246)
	Central IRB	137 (73.3%)	162 (129, 200)
	Local IRB	47 (25.1%)	264 (207, 439)
	Missing IRB	3 (1.6%)	222 (215, 451)
ODYSSEY	All sites	285	292 (203, 405)
	Central IRB	169 (59.3%)	249 (179, 336)
	Local IRB	116 (40.7%)	359 (275, 508)
EUCLID	All sites	142	280 (223, 343)
	Central IRB	89 (62.7%)	252 (198, 301)
	Local IRB	53 (37.3%)	329 (278, 400)

Continuous variables are described using median (25th, 75th percentile). Categorical variables are described using frequency (%). IRB = institutional review board.

© 2021 Goyal A et al. *JAMA Network Open.*
